# Validation of a Novel Coronary Angiography-Derived Quantitative Functional Assessment Compared with Wire-Based FFR and IMR: The Prospective Multicenter FAIR Study

**DOI:** 10.3390/jcm14134503

**Published:** 2025-06-25

**Authors:** Changwu Xu, Qiang Xue, Jianwen Liang, Guosheng Fu, Qiang Wu, Qing Jin, Wenbin Wei, Fuyu Qiu, Huali Yao, Hong Jiang

**Affiliations:** 1 Department of Cardiology, Renmin Hospital of Wuhan University, Wuhan 430060, China; xuchangwu@whu.edu.cn; 2 Department of Cardiology, Yan’an Hospital of Kunming City (Yan’an Hospital Affiliated to Kunming Medical University), Kunming 650051, China; xueqiang@kmmu.edu.cn (Q.X.); jinqing@kmmu.edu.cn (Q.J.); 3 Department of Cardiology, The Eighth Affiliated Hospital, Sun Yat-sen University, Shenzhen 518033, China; liangjw39@mail.sysu.edu.cn (J.L.); weiwb@mail.sysu.edu.cn (W.W.); 4 Department of Cardiology, Sir Run Run Shaw Hospital, Zhejiang University School of Medicine, Hangzhou 311500, China; fugs@zju.edu.cn (G.F.); 3309029@zju.edu.cn (F.Q.); 5 Department of Cardiology, Jieyang People’s Hospital, Jieyang 522091, China; wqxxx158@163.com (Q.W.); yaohuali_865@163.com (H.Y.)

**Keywords:** myocardial ischemia, fractional flow reserve, index of microcirculatory resistance, patient-specific pressure

## Abstract

**Background:** Synchronous computation of coronary angiography-derived fractional flow reserve (CAG-FFR) and coronary angiography-derived index of microcirculatory resistance (CAG-IMR) is a novel coronary angiography-based method for on-site assessment of suspected myocardial ischemia in patients with coronary artery disease (CAD). **Methods:** This trial is a prospective, multicenter, controlled study designed to assess the diagnostic performance of CAG-FFR and CAG-IMR in patients with suspected myocardial ischemia using wire-based FFR and IMR as reference standards. The functional parameters were calculated using a reduced order computational fluid dynamics solver that incorporates thrombolysis in myocardial infarction (TIMI) frame count and aortic pressure recorded by a disposable invasive pressure sensor. **Results:** CAG-FFR was computed in 325 patients, demonstrating a patient-level diagnostic accuracy of 95.4%, sensitivity of 95.9%, and specificity of 95.1%. The area under the receiver operating characteristic curve (AUC) of CAG-FFR was 0.977. Patient-specific aortic pressure adoption significantly improved the accuracy of CAG-FFR in the “gray zone” compared to fixed-pressure models. In addition, CAG-IMR was successfully computed in 180 patients, showing a patient-level diagnostic accuracy of 95.5%, sensitivity of 96.4%, and specificity of 95.2%. The AUC of CAG-IMR in diagnosing abnormal coronary microcirculatory dysfunction was 0.973. **Conclusions:** Synchronous computation of CAG-FFR and CAG-IMR demonstrated higher feasibility and excellent diagnostic accuracy compared to wire-based FFR and IMR, highlighting its clinical potential for CAD evaluation.

## 1. Introduction

Coronary artery disease (CAD) is the leading cause of cardiovascular mortality and morbidity worldwide [1]. Intracoronary physiological assessment is acknowledged as a valuable strategy to identify the presence of myocardial ischemia (MI) in patients with CAD, serving as both a crucial determinant of clinical prognosis and a foundation for rational and effective treatment [2]. MI is primarily caused by epicardial stenosis or microcirculatory dysfunction. Fractional flow reserve (FFR) is widely recognized as the gold standard for diagnosing MI caused by epicardial lesions [3], and the 2018 European Society of Cardiology (ESC)/European Association for Cardio-Thoracic Surgery (EACTS) guidelines on myocardial revascularization specifically recommend FFR for evaluating the hemodynamic significance of intermediate-grade coronary stenoses when ischemia is not attributable to other etiologies [4]. However, its diagnostic accuracy is limited in patients with coronary microvascular dysfunction (CMD), which occurs independently of epicardial stenosis [5,6]. Relevant studies indicated that the index of microcirculatory resistance (IMR), as an independent predictor [5,7], is valuable in assessing MI and widely used clinically in patients with CMD. Meanwhile, the clinical application of wire-based FFR and IMR is significantly restricted in scenarios involving adenosine intolerance, challenging guidewire passage, and multivessel lesions [8,9]. Simultaneous monitoring of FFR and IMR is crucial for accurately assessing the severity of epicardial coronary artery lesions and the status of microcirculation. However, conventional invasive measurement methods for these indices are complex, time-consuming, discomforting for patients, and costly, limiting their widespread clinical application [10,11,12].

Recent advancements in medical image processing technology have led to significant breakthroughs in functional assessment techniques using angiographic images. Since its introduction in 2013, FFR calculation based on coronary angiography has been developed significantly over the past decade [13,14]. Building on this progress, the estimation of IMR from angiographic images has also emerged as a prominent area of research in recent years [15,16,17]. Previous studies indicated that non-invasive FFR and IMR derived from angiography images rival the diagnostic accuracy of invasive methods [15,18,19]. Simulation-based solutions, recognized and recommended by clinical guidelines and expert consensus, offer significant potential by co-registering physiological assessments with imaging techniques [20,21]. However, the current methods have several limitations. First, only one vessel is reconstructed in a three-dimensional (3D) model, which prevents the evaluation of multiple lesions in different vessels simultaneously. Second, previous study introduced empirical formulas for calculating pressure drop to address the issue of time-consuming computation in traditional computational fluid dynamics (CFD) methods [22]. This simplified way can yield results within seconds but with less accuracy, as it only considers the radius and length of vessels while neglecting other key factors, such as vessel curvature and flow profile. In addition, using fixed aortic pressure as a boundary condition inevitably compromises accuracy, particularly for patients in the FFR “gray zone”. Finally, as mentioned above, no established approaches currently can simultaneously and non-invasively provide both FFR and IMR, despite the recognized importance and critical need for both indices in comprehensive assessments. To address these limitations, we developed a novel deep learning integrated framework that supports multi-branch model reconstruction and fast simultaneous computation of coronary angiography-derived fractional flow reserve (CAG-FFR) and coronary angiography-derived index of microcirculatory resistance (CAG-IMR). This study evaluates the diagnosis performance of these two novel indices using invasive pressure wire-based FFR and IMR as reference standards.

## 2. Material and Methods

### 2.1. Study Design and Population

The current prospective multicenter FAIR (Angiography-Derived Quantitative Functional Assessment versus Pressure-Derived FFR and IMR) study (NCT06039748) was conducted at five hospitals in China. All participating centers obtained approval from their respective ethics committees, ensuring compliance with ethical norms, and all subjects signed informed consent forms, safeguarding their rights and the legitimacy of the research. To ensure consistency across centers, all investigators underwent centralized training on study protocols, imaging acquisition standards, inclusion/exclusion criteria, and other relevant operational guidelines. A standardized case report form was utilized for data collection, and a central monitoring team perform scheduled audits to ensure protocol compliance.

This study enrolled patients aged 18 years or older who were suspected of or confirmed to have CAD and scheduled for coronary angiography. Inclusion criteria included the presence of at least one lesion with 30% to 90% diameter stenosis and reference vessel size ≥ 2 mm at the stenotic segment. Exclusion criteria were established to ensure the safety and validity of the study and included prior coronary artery bypass surgery, myocardial infarction within 7 days, NYHA functional class of III or above, serum creatinine level ≥ 150 µmol/L, allergy to iodinated contrast media or adenosine/adenosine triphosphate (ATP), and any other conditions deemed by the investigator unsuitable for FFR and IMR testing.

Furthermore, stringent imaging exclusion criteria were applied to exclude cases with target lesions involving myocardial bridges, coronary artery fistulas, or a target lesion with ≥50% diameter stenosis in the left main coronary artery or right coronary artery (RCA) less than 3 mm to the ostium. Additionally, cases with poor contrast filling resulting in indistinct vessel boundaries, severe vessel overlap, or severe target vessel tortuosity that prevented complete identification of the lesion location were also excluded from enrollment.

### 2.2. Methodology for CAG-FFR and CAG-IMR Determination Using Multi-Branch CFD Simulation

Computation of CAG-FFR and CAG-IMR were performed using a dedicated analysis system (AngioQFA, Raysight Medical Co., Ltd., Shenzhen, China) by an experienced analyst blinded to wire-based FFR and IMR measurements. The analysis workflow involves multi-branch model reconstruction followed by CFD simulations. At least two angiographic images with an angular separation of ≥25° are required for initial 3D reconstruction. This selection strategy ensures comprehensive capture of vascular anatomy, providing a robust basis for subsequent CFD computation and diagnostic evaluation. The simulation process incorporates the determination of boundary conditions and pressure calculations. Boundary conditions include the inlet pressure of the coronary artery and the mean flow rate. Aortic blood pressure is measured using a high-precision disposable transducer that transmits real-time data to the software system. The total mean volumetric flow rate is estimated by dividing the volume of the reconstructed coronary artery model by the flow transport time derived from thrombolysis in myocardial infarction (TIMI) frame counts [23,24].

Each reconstructed vessel is automatically divided into segments, with flow rates assigned to each. The flow rate for each segment is determined based on the reference diameter and the spatial distribution of side branches. The reference diameter was determined by the approach described in the literature [25]. The flow rate decreases proportionally with changes in the reference diameter. At bifurcations, the flow rate distribution is determined based on the cross-sectional areas of the two branches.

The pressure in each segment is calculated by solving the modified Bernoulli equation:

(1) $$\frac{P_{1}}{\rho g}+\frac{\alpha_{1}E_{1}}{g}=\frac{P_{2}}{\rho g}+\frac{\alpha_{2}E_{2}}{g}+∆h$$

where ∆h represents hydraulic loss, determined by the segment’s flow rate (q) and total resistance (r). Here, P denotes pressure, α is the kinetic energy correction coefficient, and E represents blood flow kinetic energy.

After completing these steps, velocity and pressure distributions across the multi-branch coronary artery model are obtained. CAG-FFR at any location is then computed as follows:

(2) $$CAG-FFR=\frac{P_{d}}{P_{a}}$$

where P_d_ is the local pressure, and P_a_ is the pressure at the coronary ostium. The spatial distribution of CAG-FFR values is visualized.

For the $k_{th}$ branch, the CAG-IMR can be calculated as follows:

(3) $$CAG-{IMR}_{k}=P_{k}\times T_{mn}$$

where $P_{k}$ represents the distal pressure of the $k_{th}$ branch. $T_{mn}$ represents the mean transit time defined as follows:

(4) $$T_{mn}=\sum_{i=1}^{n}\frac{L_{i}}{V_{i}}$$

where L denotes the length of segment i, and V represents the blood flow velocity at that segment. By summing the flow transit time for each vascular branch, we can obtain a more precise $T_{mn}$. The methodology for CAG-IMR calculation is detailed in prior research [26]. Notably, the velocity and Pa in the above equations were adjusted to their corresponding values under hyperemic conditions, as described in a previous study [27].

### 2.3. Strategies for Multi-Branch Model Reconstruction and Fast Simulation

**Figure 1:**
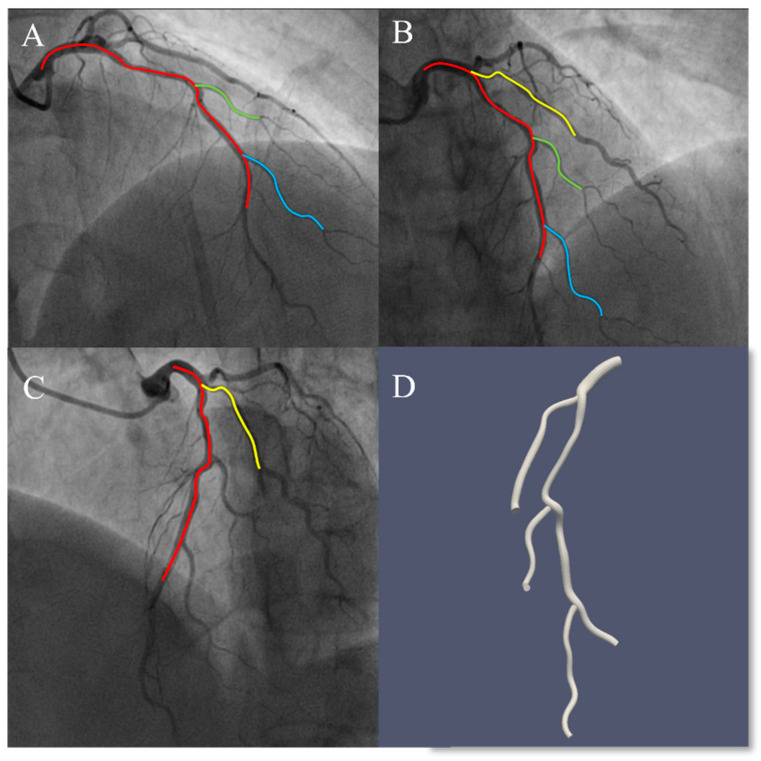
Multi-branch reconstruction with the CSS technique. (**A**) LAD, D2, and D3 are available for reconstruction. (**B**) LAD, D1, D2, and D3 are available for reconstruction. (**C**) LAD and D1 are available for reconstruction. LAD can be reconstructed by any 2 sequences from (**A**–**C**). D1 can be reconstructed by (**B**,**C**). D2 and D3 can be reconstructed by (**A**,**B**). (**D**) A 3D multi-branch model.

This study introduces several new strategies in the analysis workflow to improve the completeness of coronary artery tree modeling. A key advancement in the AngioQFA system is the implementation of multi-view integration framework, which enables the reconstruction of complex vascular branching patterns. This framework incorporates one to two additional angiographic projections with distinct angular orientations into the existing dataset, thereby addressing the limitations of double-view imaging in capturing completeness of vascular architecture. As demonstrated in Figure 1, an operator can reconstruct the left anterior descending artery (LAD) and its side branches (D2, D3) using images A and B and then incorporate image C to reconstruct the D1 branch. This approach allows simultaneous analysis of multiple lesions across different vessels in a single patient.

Traditional 3D CFD solvers are computationally intensive due to mesh generation and Navier–Stokes equation resolution, limiting real-time clinical application. To address this, an efficient 0D solver was developed that is capable of computing pressure fields within seconds, enabling the entire analysis to be completed in minutes. The solver incorporates an adaptive parameter optimization strategy that integrates multidimensional anatomical and hemodynamic features—including vessel diameter, length, curvature, and upstream flow characteristics—into resistance calculations. Specifically, these parameters are systematically embedded using a dynamic algorithm that adjusts resistance coefficients in Equation (1) according to segment-specific characteristics. This data-driven enhancement maintains high computational efficiency while improving accuracy in pressure estimation.

### 2.4. Wire-Based FFR and IMR Measurements

**Figure 2:**
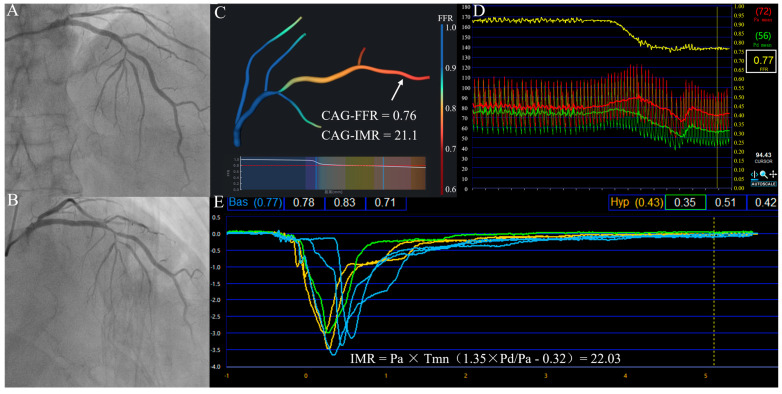
(**A**,**B**) Angiography images at different projection angles. (**C**) A 3D multi-branch model and functional results. (**D**) Wire-based FFR measurement. (**E**) Wire-based IMR measurement.

FFR was measured in all cases using the RadiAnalyzer Xpress device (St. Jude Medical, Uppsala, Sweden) and a coronary pressure wire (Certus, St. Jude Medical). After calibration and equalization, the pressure wire was advanced distally to the stenosis until the pressure sensor landed in a smooth coronary segment. Hyperemia was induced by the administration of intravenous (140 μg/kg/min) or intracoronary (at least 100 μg) adenosine or ATP. After measurement, the pressure sensor was returned to the guiding catheter tip to check whether pressure drift existed. Figure 2 shows the variation in pressure and the FFR curve during measurement.

IMR was measured with the same system using the established thermodilution technique. The procedures followed the standard guidelines recommended by the RadiAnalyzer Xpress instrument [28]. As the system can only provide the flow transit time, IMR must be calculated manually using the following equation [29]:

(5) $${\mathrm{IMR}}_{\mathrm{wire}}=P_{d}\times T_{mn}$$

or follow the modified equation for those patients with severe epicardial stenosis [30]:

(6) $${\mathrm{IMR}}_{\mathrm{wire}}=P_{a}\times T_{mn}(1.35*\frac{P_{d}}{P_{a}}-0.32)$$

where $T_{mn}$ is the mean transport time at hyperemia, which can be obtained from the display interface (Figure 2E).

### 2.5. Study Endpoints

The primary endpoints were the sensitivity and specificity of CAG-FFR and CAG-IMR using wire-based FFR and IMR as reference standards with a clinical significance cut-off value of 0.80 for FFR and 25U for IMR, respectively. Secondary endpoints included diagnostic accuracy, positive predictive value (PPV), negative predictive value (NPV), agreement, correlation, and the area under the receiver operating characteristic curve (AUC) for CAG-FFR versus FFR and CAG-IMR versus IMR.

### 2.6. Sample Size

The performance goals of the coprimary end points for both CAG-FFR and CAG-IMR were assumed to be a sensitivity = 93% and specificity = 90%. The corresponding target values of sensitivity and specificity were set as 83% and 80% for CAG-FFR and 76% and 76% for CAG-IMR, respectively. With an assumed prevalence of positive FFR as 33% to 50%, type I error as 0.025 (1-sided), statistical power as 90%, and an expected dropout rate as 10%, the total enrollment numbers for the FFR and IMR groups were at least 330 and 144 patients, respectively. All the patients undergoing IMR measurement were sourced from the FFR enrollment cohort. From the beginning of enrollment, both FFR and IMR were performed for each enrolled patient, until the enrollment number for the IMR group met its requirement. Thereafter, only FFR was measured for the remaining enrolled patients.

### 2.7. Intra- and Inter-Observer Analysis

To validate intra-observer and inter-observer variability in CAG-FFR and CAG-IMR analysis, 50 patients were randomly selected and reanalyzed by the same analyst 1 week later and by the second analyst. The procedure performed in the second analysis or by the second analyst was blinded to the previous results.

### 2.8. Statistical Analyses

Continuous variables were summarized as mean ± standard deviation, while categorical variables were presented as counts and percentages. We employed Spearman’s correlation coefficient to examine the relationships between variables and linear regression analysis to quantify these associations. Bland–Altman analysis was performed to assess the agreement between computational and wire-based indicators. The AUCs were calculated for CAG-FFR and CAG-IMR, with a reference standard of wire-based FFR ≤ 0.8 and IMR ≥ 25U [31]. Diagnostic performance of CAG-FFR was evaluated in subgroups, including patients and vessels categorized by visual degree of stenosis and FFR “gray zone” lesions (0.75 ≤ FFR ≤ 0.85). Likewise, diagnostic performance of CAG-IMR was also evaluated in subgroups, including patients and vessels categorized by visual degree of stenosis and significant epicardial stenosis (FFR ≤ 0.8). Significance was established at a *p*-value < 0.05, with all analyses performed using SciPy package in Python 3.12.0.

## 3. Results

### 3.1. Patient Characteristics

**Figure 3:**
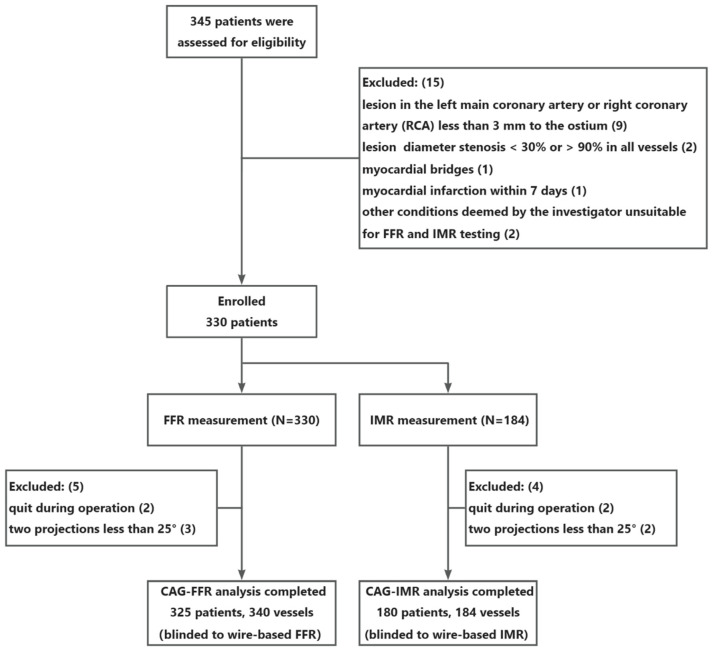
Flowchart of patient enrollment. FFR, fractional flow reserve; IMR, index of microcirculatory resistance.

A total of 345 patients were assessed for eligibility across five centers between September 2023 and January 2024. Within the FFR assessment cohort, fifteen subjects were excluded due to the following reasons: ostial lesion (9), lesion diameter stenosis < 30% or >90% in all vessels (2), myocardial bridges (1), MI within 7 days (1), and unsuitable for FFR or IMR testing (2). In total, 330 patients were successfully enrolled for FFR measurement, and five subjects were excluded due to unsatisfied imaging projection angle or failing to measure the aortic pressure using a disposable pressure sensor during the operation, resulting in a final enrollment of 325 patients, involving 340 coronary vessels (Figure 3). Baseline clinical characteristics of the FFR group are summarized in Table 1. Patients averaged 62.5 years of age, and 67.4% were male. Other characteristics included average body mass index (BMI, 24.6), hypertension (68.6%) and diabetes (33.2%). Among the 340 vessels analyzed, the LAD was the most frequently involved (67.6%), followed by the RCA (21.2%), and the left circumflex artery (LCX, 11.2%). Notably, the cohort included 50 patients with a documented history of myocardial infarction, 36 of whom had received percutaneous coronary intervention (PCI) before study enrollment. This demographic and clinical profile serves as the basis for the subsequent analysis of the trial outcomes.

Among these 330 cases, 184 cases were included in the IMR evaluation. Two patients failed to complete the measurement due to unexpected circumstances during the process, and two were excluded due to the limitation in imaging projection. Consequently, 180 patients with 184 vessels were enrolled in the final IMR analysis cohort. Baseline characteristics of the IMR group are shown in Table 2. It is noteworthy that among individuals with FFR > 0.8, 31.3% suffered from microcirculatory dysfunction (IMR ≥ 25U). Similarly, in patients with FFR ≤ 0.8, the prevalence of microcirculatory dysfunction was 27.3%. This distribution underscores the fact that the presence of CMD is not directly correlated with the degree of epicardial stenosis.

### 3.2. Diagnostic Performance of CAG-FFR

**Figure 4:**
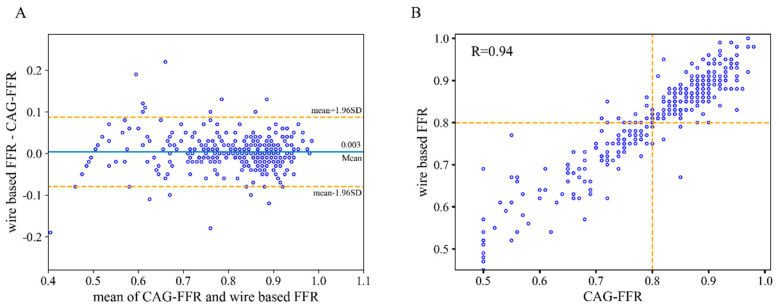
Comparison of CAG-FFR and wire-based FFR. (**A**) Bland–Altman plot of FFR and CAG-FFR on a per-patient basis. (**B**) Scatterplot of wire based FFR and CAG-FFR. R represents the Pearson’s coefficient.

As shown in Table 3, the results demonstrated the outstanding diagnostic performance of CAG-FFR, with an overall accuracy of 95.4% at the patient level. Specifically, the sensitivity (95.9%), specificity (95.1%), PPV (92.1%), and NPV (97.5%) all demonstrated high reliability. The CAG-FFR showed significantly better performance than that with visual degree of stenosis (DS) by 70% or 50%. Using Bland–Altman plot analysis, the agreement between CAG-FFR and the wire-based FFR measurements reached a high level, with a strong correlation coefficient of 0.94 (Figure 4). In addition, a higher AUC was observed as compared with DS for per-patient discrimination (0.977 vs. 0.845). Despite a slight decrease in diagnostic performance at the vessel level, it remains at a high standard, with an accuracy, sensitivity, specificity, PPV, and NPV of 94.7%, 93.7%, 95.31%, 92.3%, and 96.2%, respectively. Furthermore, we compared the results with CAG-FFR computed using the fixed pressure of 94 mmHg (mean pressure at rest [32]) as adopted in other studies. It was found that the accuracy decreased to 91.4%, while sensitivity and specificity decreased to 90.2% and 92.1%, respectively.

In the wired FFR and DS subgroup analysis, we focused on those cases in the “gray zone” (0.75 ≤ FFR ≤ 0.85) and the intermediate lesions (50% ≤ DS < 70%) in vessel level. The accuracy is 86.1% in the “gray zone” group and 95.4% in vessels with intermediate lesions. Of note, the accuracy significantly decreases to 76.1% for those patients with 0.75 ≤ FFR ≤ 0.85, with a sensitivity and specificity of 77.1% and 75.4%, respectively, when a fixed pressure was adopted. The detailed performance of the subgroup analysis is shown in Tables S1 and S2 in the Supplementary Materials.

### 3.3. Diagnostic Performance of CAG-IMR

**Figure 5:**
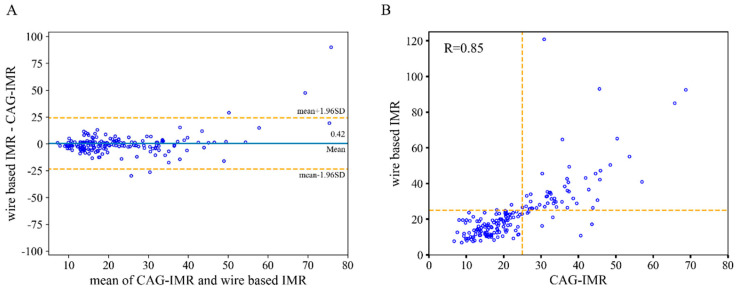
Comparison of CAG-IMR and wire-based IMR. (**A**) Bland–Altman plot of IMR and CAG-IMR on a per-patient basis. (**B**) Scatterplot of wire based IMR and CAG-IMR. R represents the Pearson’s coefficient.

**Figure 6:**
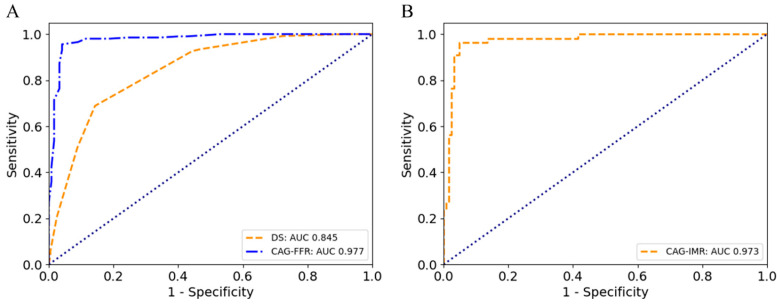
(**A**) AUC of CAG-FFR versus DS (visual diameter stenosis in ICA) for demonstration of ischemia (FFR ≤ 0.80) on a per-patient basis. (**B**) AUC of CAG-IMR for demonstration of microcirculatory dysfunction (IMR ≥ 25U) on a per-patient basis.

As shown in Table 4, this study also found that CAG-IMR demonstrated exceptional diagnostic accuracy, reaching 95.5% at the patient level. In terms of specific indicators, the sensitivity (96.4%), specificity (95.2%), PPV (89.8%), and NPV (98.4%) all demonstrated high clinical value. Using Bland–Altman plot analysis, the agreement between CAG-IMR and wire-based IMR measurements reached an excellent level, with a correlation coefficient of 0.85 (Figure 5). Remarkably, CAG-IMR yielded an outstanding AUC value of 0.973 (Figure 6). At the vessel level, CAG-IMR showed excellent performance compared with wire-based measurements, with accuracy, sensitivity, specificity, PPV, and NPV reaching values as high as 95.6%, 96.4%, 95.4%, 89.8%, and 98.4%, respectively. Similar to CAG-FFR, the accuracy of CAG-IMR decreased to 89.4%, accompanied by a decrease in sensitivity and specificity to 85.5% and 91.2%, respectively, when patient-specific aortic pressure was replaced by the fixed pressure in the computation (Table S3 in the Supplementary Materials).

We performed further sensitivity analyses to evaluate the diagnostic performance of CAG-IMR in groups with FFR ≤ 0.8 and FFR > 0.8. The accuracy was 95.8% and 95.5%, respectively, at the patient level (Table 4), with no significant difference between these two groups (*p* = 0.802). In addition, the performance of CAG-IMR in patients with different DS was also investigated. The accuracy remained comparable (100% in group with DS < 50%, 93.4% in group with 50% ≤ DS < 70%, 96.4% in group with 70% ≤ DS ≤ 90%), and there was no statistical difference among them (*p* = 0.486). The detailed data are provided in Table S4 in the Supplementary Materials.

### 3.4. Reproducibility in Repeated CAG-FFR and CAG-IMR Analysis

Repeated CAG-FFR and CAG-IMR analysis was performed in 50 vessels. Intra-observer and inter-observer variability in CAG-FFR was 0.00 ± 0.03 and 0.00 ± 0.04, respectively. Likewise, the intra-observer and inter-observer variability in CAG-IMR was −0.12 ± 2.13 and −0.44 ± 3.86, respectively.

## 4. Discussion

This trial employed wire-based FFR and IMR as reference standards for a head-to-head validation of the diagnostic performance of CAG-FFR and CAG-IMR in assessing MI. The results confirmed a comparable diagnostic accuracy of CAG-FFR and CAG-IMR and validated the feasibility of simultaneous assessment of epicardial vessel ischemia and microcirculatory function. The streamlined and efficient measurement process provided important practical insights into the real-world applicability of this technology, which is poised for broader recognition and accessibility in clinical settings.

### 4.1. Functional Coronary Angiography of FFR and IMR

In this study, we achieved functional coronary angiography (FCA) of FFR and IMR using CAG-FFR and CAG-IMR. FCA, a novel modality for assessing the physiology of coronary obstructions, has been employed worldwide for CAD in various clinical settings [21]. In comparison to traditional invasive detection methods, FCA does not require a pressure wire or pharmacological hyperemia, thus potentially reducing the burden of cost, time, and complication risks associated with the invasive means, thereby opening a safer and more economical avenue for a diagnostic pathway for CAD patients [21,33].

Previous studies have demonstrated that computational techniques for FFR estimation based on angiographic images, such as QFR, vFFR, caFFR, and FFR_angio_, exhibit robust diagnostic performance with accuracies generally exceeding 90% [22,34,35,36]. This study further solidifies the reliability of these FCA assessment techniques in terms of diagnosis performance. Notably, CAG-IMR, as a new FCA for evaluating microcirculatory function, has also demonstrated exceptional diagnostic capabilities, rivaling the performance of similar products. Other angiography-based methods of deriving IMR also showed a good correlation with invasively measured IMR [15,16,37,38]. These findings collectively suggest that this approach could provide physicians with important insights into the microcirculatory properties downstream of the examined coronary artery. This has significant implications for early detection and intervention of microcirculatory impairments, ultimately improving patient outcomes.

### 4.2. Innovations in Coronary Vessel Modeling

The conventional angiography-based FFR technologies mentioned above are limited to reconstructing a single-vessel model without side branches by selecting only two images. In contrast, the innovation of this study lies in the precise reconstruction of coronary vessel branch structures within a 3D model. The current framework effectively overcomes the challenges posed by vessel curvature and overlapping structures by leveraging multiple image sequences or different frames within the same sequence. This strategy improves the success rate of model construction, ensures the accuracy of flow distribution simulations, and therefore strengthens its applicability in assessing complex lesions. In particular, compared to 2D reconstruction methods, the 3D multi-branch model employed in this study showed clear advantages in dealing with eccentric lesions and highly tortuous vessels.

### 4.3. Impact of Aortic Pressure on Diagnostic Accuracy

The specific value of aortic pressure has a significant impact on the accuracy of CAG-FFR and CAG-IMR calculations, particularly in individuals in the gray zone. Utilizing a fixed aortic pressure adversely influences the results. We observed that when a fixed blood pressure of 94 mmHg was adopted, the accuracy of both CAG-FFR and CAG-IMR decreased, which is consistent with previous study [39]. Of note, the accuracy of CAG-FFR significantly decreases to 76.1% for those patients with 0.75 ≤ FFR ≤ 0.85. Therefore, we incorporate a disposable pressure sensor in the AngioQFA system to acquire precise pressure measurements and avoid assignment of a fixed value.

### 4.4. Microvascular Dysfunction in Patients Without Significant Stenosis

Previous studies have indicated that about 20~50% of patients with MI do not exhibit epicardial flow-limiting stenosis but typically suffer from CMD [40,41]. Therefore, our investigation paid particular attention to the prevalence of microvascular disease among patients without significant epicardial artery stenosis (FFR > 0.8). The result indicated the percent of patients who suffer from CMD is close in both groups (31.3% in patients with FFR > 0.8 vs. 27.3% in patients with FFR ≤ 0.8). In clinical practice, physicians may adopt an entirely different treatment strategy based on the microvascular function status [42,43]. IMR plays a pivotal role in identifying whether microcirculation is the leading cause of symptoms, as well as predicting periprocedural events and adverse outcomes for percutaneous coronary intervention (PCI) [7,44,45], and guiding adjunctive therapy [46]. For example, persistent symptoms following PCI may often be attributed to underlying CMD, which necessitates substantially tailored treatment strategies [42,47]. Our study further confirmed and underscored the importance of a comprehensive assessment of both epicardial and microvascular function, which is another innovation of the AngioQFA system as the previous techniques mentioned above could not provide FFR and IMR in one analysis. The comparable performance of CAG-IMR in patients with or without epicardial flow-limiting stenosis reveals its enormous promise in auxiliary diagnosis. Furthermore, the screening of microvascular disease is particularly crucial in scenarios where symptoms persist after PCI or ischemic indications remain unresolved, as microvascular dysfunction in this subgroup of patients may be the underlying cause of persistent symptoms and a high risk of cardiovascular events [17,48,49].

### 4.5. Clinical Implications

From a clinical application perspective, the precise and integrated FCA proposed in this study could provide a powerful diagnostic tool with its efficient, convenient, and cost-effective characteristics. The novel approach enables the simultaneous evaluation of epicardial stenosis and microvascular function within several minutes, thereby circumventing the need for complex, expensive, and potentially risky invasive measurements. Furthermore, the methodology’s rapid analysis within about 5 min places minimal burden on the catheterization lab, facilitating real-time diagnosis and decision-making. More important, by integrating functional assessment into standard angiographic workflows, it addresses critical diagnostic uncertainties—particularly in patients with intermediate coronary stenosis (diameter stenosis 50–70%), patients referred for coronary angiography following a positive functional test or CTA, and those falling within the FFR gray zone (0.75–0.85), where conventional anatomical criteria alone are insufficient to guide revascularization decisions. This has profound implications for advancing the precision and efficiency of CAD management.

To realize the broader clinical application of angiography-derived functional indices, two critical challenges must be addressed. First, the robustness of the entire analytical framework must be enhanced to minimize operator dependency, ensuring consistent and reproducible results across diverse clinical settings. This includes optimizing image preprocessing algorithms, integrating machine learning for automated lesion identification, and implementing automated flow velocity estimation. Second, establishing a strong correlation between these indices and long-term clinical outcomes is essential to demonstrate their prognostic value. Resolving these challenges would position angiography-derived non-invasive functional indices as viable alternatives to invasive functional assessments, accelerating their integration into routine clinical practice.

## 5. Limitations

This study has several limitations. First, we did not specifically control for the inclusion proportion of side branch lesion. As a result, only four cases with side-branch involvement were evaluated with wire-based FFR and IMR, highlighting the need for a larger population of side branch lesions to validate the framework’s robustness in these scenarios. Second, CAG-IMR was derived from angiography-derived diastolic flow velocity at rest, which differs from wire-based IMR assessed during maximal hyperemia. Third, although the analysis was conducted by an experienced operator, individual variability in TIMI frame counting was inevitable, thus introducing errors particularly in those cases with poor image quality. Therefore, adopting an automated method could offer a more reliable alternative in the future.

## 6. Conclusions

This prospective, multicenter, controlled clinical trial evaluated the diagnostic performance of two angiography-derived functional parameters, namely CAG-FFR and CAG-IMR. The findings successfully demonstrated that both indicators show high diagnostic performance in identifying myocardial ischemia. The innovative computational approach derives physiology directly from anatomy, integrates sufficient patient-specific information, and enables the simultaneous evaluation of coronary lesion anatomy and a thorough assessment of coronary physiology within a streamlined and intuitive system offering accurate physiological insights. These advancements highlight the potential of CAG-FFR and CAG-IMR as reliable, efficient tools for guiding clinical decision-making in CAD management.

## Figures and Tables

**Table 1:** Baseline characteristics of patients in the FFR group.

Patients		Total N = 325
Age, year		62.5 ± 9.6
Male		219 (67.4)
BMI, kg/m^2^		24.6 ± 3.2
Hypertension		223 (68.6)
Hyperlipidemia		120 (36.9)
Diabetes		108 (33.2)
Family with CAD		7 (2.15)
Medication	Heparin sodium injection	89 (27.4)
	Rosuvastatin calcium tablets	56 (17.2)
	Aspirin enteric-coated tablets	62 (19.1)
	Metoprolol succinate sustained-release tablets	61 (18.7)
	Clopidogrel hydrogen sulfate tablets	83 (25.5)
	Nitroglycerin	38 (11.7)
Previous myocardial infarction (%)		Total N = 50 (15.38)
PCI of culprit lesion		36 (72)
Multi vessel disease		38 (76)
INOCA		5 (10)
Symptoms	Stable angina	21 (42)
	Unstable angina	20 (40)
	Asymptomatic	9 (18)
Lesions		Total N = 340
LAD		230 (67.6)
RCA		72 (21.2)
LCX		38 (11.2)
Diameter stenosis, %		67.6 ± 12.4
FFR		0.808 ± 0.117
Vessels with FFR ≤ 0.8		128 (37.6)

Values are mean ± SD, or n (%); SD, standard deviation; BMI, body mass index; CAD, coronary artery disease; LAD, left anterior descending artery; RCA, right coronary artery; LCX, left circumflex artery; FFR, fractional flow reserve.

**Table 2:** Baseline characteristics of patients in the IMR group.

Patients		Total N = 180
Age, year		62.2 ± 9.5
Male		127 (70.6)
BMI, kg/m^2^		24.8 ± 3.2
Hypertension		223 (68.6)
Hyperlipidemia		74 (41.1)
Diabetes		56 (31.1)
Family with CAD		7 (3.9)
Medication	Heparin sodium injection	42 (23.3)
	Rosuvastatin calcium tablets	47 (26.1)
	Aspirin enteric-coated tablets	49 (27.2)
	Metoprolol succinate sustained-release tablets	50 (27.8)
	Clopidogrel hydrogen sulfate tablets	66 (36.7)
	Nitroglycerin	14 (7.8)
Previous myocardial infarction		Total N = 29 (16.1)
PCI of culprit lesion		20 (69.0)
Multi vessel disease		21 (72.4)
INOCA		5 (17.2)
Symptoms	Stable angina	9 (31.0)
	Unstable angina	14 (48.3)
	Asymptomatic	6 (20.7)
Lesions		Total N = 184
LAD		129 (70.1)
RCA		39 (21.2)
LCX		16 (8.7)
Diameter stenosis, %		66.1 ± 12.5
IMR		23.8 ± 21.4
Vessels with IMR ≥ 25U		55 (29.9)
Vessels with FFR ≤ 0.8		66 (35.9)

Values are mean ± SD, or n (%); SD, standard deviation; BMI, body mass index; CAD, coronary artery disease; LAD, left anterior descending artery; RCA, right coronary artery; LCX, left circumflex artery; FFR, fractional flow reserve; IMR, index of microcirculatory resistance.

**Table 3:** Per-patient diagnostic performance of CAG-FFR and ICA.

	CAG-FFR ≤ 0.8	With DS ≥ 50%	With DS ≥ 70%
Accuracy, % (95% CI)	95.4 (92.5–97.4)	42.5 (37.0–48.0)	68.8 (63.6–73.7)
Sensitivity, % (95% CI)	95.9 (90.7–98.6)	100.0 (97.0–100.0)	92.9 (86.9–96.7)
Specificity, % (95% CI)	95.1 (91.1–97.6)	7.9 (4.6–12.5)	54.5 (47.5–61.3)
PPV, % (95% CI)	92.1 (85.9–96.1)	39.5 (34.0–45.2)	54.9 (48.0–61.7)
NPV, % (95% CI)	97.5 (94.2–99.1)	100.0 (79.5–100.0)	92.8 (86.7–96.6)

ICA, invasive coronary angiography; DS, diameter stenosis; CI, confidence interval; PPV, positive predictive value; NPV, negative predictive value.

**Table 4:** Per-patient diagnostic performance of CAG-IMR.

	CAG-IMR ≥ 25U	With FFR > 0.8	With FFR ≤ 0.8
Accuracy, % (95% CI)	95.5 (91.4–98.1)	95.8 (90.4–98.6)	95.5 (87.3–99.1)
Sensitivity, % (95% CI)	96.4 (87.5–99.6)	94.6 (81.8–99.3)	100 (81.5–100.0)
Specificity, % (95% CI)	95.2 (89.9–98.2)	96.3 (89.6–99.2)	93.8 (82.8–98.7)
PPV, % (95% CI)	89.8 (82.1–97.5)	92.1 (78.6–98.3)	85.7 (63.7–97.0)
NPV, % (95% CI)	98.4 (94.2–99.8)	97.5 (91.3–99.7)	100 (92.1–100.0)

FFR, fractional flow reserve; CI, confidence interval; PPV, positive predictive value; NPV, negative predictive value.

## Data Availability

The data that support the findings of this study are not publicly available due to ethical and privacy restrictions. However, they can be obtained from the corresponding author upon reasonable request and with the approval of the institutional ethics committee.

## Supplementary Materials

The following supporting information can be downloaded at: https://www.mdpi.com/article/10.3390/jcm14134503/s1, Table S1. CAG-FFR and ICA diagnostic performance in sub-group (vessel level); Table S2. Comparison of CAG-FFR diagnostic performance with patient-specific pressure and fixed pressure (patient level); Table S3. Comparison of CAG-IMR diagnostic performance with patient-specific pressure and fixed pressure (patient level); Table S4. CAG-IMR diagnostic performance in sub-group (vessel level).
