# Supplementary material for: Validation of a Novel Coronary Angiography-Derived Quantitative Functional Assessment Compared with Wire-Based FFR and IMR: The Prospective Multicenter FAIR Study
Source: J Clin Med. 2025 Jun 25;14(13):4503. doi: 10.3390/jcm14134503 (PMC12250030; doi:10.3390/jcm14134503)
Supplement: Supplementary file 1 [file jcm-14-04503-s001.zip › jcm-3673790-supplementary.pdf]

Validation of novel coronary angiography-derived quantitative functional assessment compared with wire-based FFR and IMR: the prospective multicenter FAIR study

Table S1. CAG-FFR and ICA diagnostic performance in sub-group (vessel level)

	FFR			DS		
	FFR < 0.75	0.75 ≤ FFR ≤ 0.85	FFR > 0.85	DS < 50%	50% ≤ DS < 69%	70 ≤ DS ≤ 90%
Accuracy, % (95% CI)	98.7 (96.1-100.0)	86.1 (79.8-92.4)	99.3 (98.0-100.0)	100.0 (100.0-100.0)	95.4 (91.4-99.3)	94.0 (90.8-97.1)
Sensitivity, % (95% CI)	98.7 (96.1-100.0)	86.5 (77.3-95.8)	Na	Na	77.8 (50.6-100.0)	95.0 (91.0-98.9)
Specificity, % (95% CI)	Na	85.7 (77.1-94.4)	99.3 (98.0-100.0)	100.0 (100.0-100.0)	97.0 (93.6-100.0)	92.7 (87.5-97.9)
PPV, % (95% CI)	100.0 (100.0-100.0)	83.3 (73.4-93.3)	0.0 (0.0-0.0)	0.0 (0.0-0.0)	70.0 (41.6-98.4)	94.2 (90.0-98.4)
NPV, % (95% CI)	0.0 (0.0-0.0)	88.5 (80.5-96.5)	100.0 (100.0-100.0)	100.0 (100.0-100.0)	98.0 (95.2-100.0)	93.7 (88.8-98.6)

ICA, invasive coronary angiography; FFR, fractional flow reserve; CI, confidence interval; PPV, positive predictive value; NPV, negative predictive value; DS, degree of stenosis(visual)

Table S2. Comparison of CAG-FFR diagnostic performance with patient-specific pressure and fixed pressure (patient level).

	Patient-specific pressure		Fixed pressure (94mmHg)	
	All patients	Patients with 0.75 ≤ FFR ≤ 0.85	All patients	Patients with 0.75 ≤ FFR ≤ 0.85
Accuracy, % (95% CI)	95.4 (92.5-97.4)	89.0 (83.1-94.9)	91.4 (88.3-94.4)	76.1 (68. -84.1)
Sensitivity, % (95% CI)	95.9 (90.7-98.6)	91.7 (83.8-99.5)	90.2 (84.9-95.4)	77.1 (65.2-89.0)
Specificity, % (95% CI)	95.1 (91.1-97.6)	86.9 (78.4-95.4)	92.1 (88.4-95.8)	75.4 (64.6-86.2)
PPV, % (95% CI)	92.1 (85.9-96.1)	84.6 (74.8-94.4)	87.3 (81.5-93.1)	71.2 (58.8-83.5)
NPV, % (95% CI)	97.5 (94.2-99.1)	93.0 (86.4-99.6)	94.0 (90.7-97.3)	80.7 (70.5-90.9)
AUC	0.977	0.888	0.969	0.836

FFR, fractional flow reserve; CI, confidence interval; PPV, positive predictive value; NPV, negative predictive value;

Table S3. Comparison of CAG-IMR diagnostic performance with patient-specific pressure and fixed pressure (patient level).

	Patient-specific pressure	Fixed pressure (94mmHg)
Accuracy, % (95% CI)	95.5 (91.4-98.1)	89.4 (85.0-93.9)
Sensitivity, % (95% CI)	96.4 (87.5-99.6)	85.5 (76.1-94.8)
Specificity, % (95% CI)	95.2 (89.9-98.2)	91.2 (86.2-96.2)
PPV, % (95% CI)	89.8 (82.1-97.5)	81.0 (70. 9-91.1)
NPV, % (95% CI)	98.4 (94.2-99.8)	93.4 (89.1-97.8)
AUC	0.973	0.956

CI, confidence interval; PPV, positive predictive value; NPV, negative predictive value;

Table S4. CAG-IMR diagnostic performance in sub-group (vessel level).

	DS < 50%	50% ≤ DS < 69%	70 ≤ DS ≤ 90%
Accuracy, % (95% CI)	100 (75.3-100.0)	93.4 (84.1-98.2)	96.4 (91.0-99.0)
Sensitivity, % (95% CI)	100 (54.1-100.0)	88.9 (65.3-98.6)	100 (88.8-100.0)
Specificity, % (95% CI)	100 (59.0-100.0)	95.3 (84.2-99.4)	94.9 (87.5-98.6)
PPV, % (95% CI)	100 (54.1-100.0)	88.9 (65.3-98.6)	88.6 (73.3-96.8)
NPV, % (95% CI)	100 (59.0-100.0)	95.3 (84.2-99.4)	100 (95.2-100.0)

CI, confidence interval; PPV, positive predictive value; NPV, negative predictive value;

DS, degree of stenosis(visual)
